# Supplementary material for: Comparison of two methods to assess physical activity prevalence in children: an observational study using a nationally representative sample of Scottish children aged 10–11 years
Source: BMJ Open. 2018 Jan 24;8(1):e018369. doi: 10.1136/bmjopen-2017-018369 (PMC5786112; doi:10.1136/bmjopen-2017-018369)
Supplement: Supplementary file 1 [file bmjopen-2017-018369supp001.pdf]

Supplementary Table 1: Comparison of weighted sample to known national level SES/demographic distributions

Demographic variable	SPACES weighted sample distribution	Sweep 8 Growing up in Scotland (GUS) weighted sample distribution
Income (per annum)		
<3,999 - £9,999	3%	5%
£10,000 - £19,999	20%	21%
£20,000 - £28,999	18%	16%
£29,000 - £37,999	14%	14%
£38,000 - £49,999	14%	15%
>50,000	27%	29%
Mothers age at birth (years)		
Under 20	4%	7%
20 -29	43%	41%
30 – 39	50%	49%
40 or older	3%	3%
Marital status		
Married	61%	68%
Cohabiting	19%	15%
Single	11%	9%
Widowed	1%	1%
Divorced	5%	3%
Separated	3%	4%
SIMD quintile (2012)		
Most	21%	20%
2nd	18%	21%
Middling	19%	20%
4th	21%	18%
Least Deprived	21%	21%
Highest educational qualification in household		
No qualification	3%	6%
Lower level Standard Grades or equivalent	3%	4%
Upper level Standard Grades or equivalent	19%	19%
Higher Grades or equivalent	39%	33%
Degree level academic or equivalent	35%	38%
Other	1%	0.4%
Urban/Rural dwelling		
Large urban	36%	38%
Other Urban	33%	32%
Small accessible towns	9%	10%
Small remote towns	3%	3%
Accessible rural	12%	13%
Remote rural	7%	4%
BMI UK categories		
Underweight	2%	2%
Healthy weight	64%	64%
Overweight	18%	15%
Obese	16%	19%
