# Supplementary material for: Comparison of two methods to assess physical activity prevalence in children: an observational study using a nationally representative sample of Scottish children aged 10–11 years
Source: BMJ Open. 2018 Jan 24;8(1):e018369. doi: 10.1136/bmjopen-2017-018369 (PMC5786112; doi:10.1136/bmjopen-2017-018369)
Supplement: Author's manuscript [file bmjopen-2017-018369.draft_revisions.pdf]

BMJ Open is committed to open peer review. As part of this commitment we make the peer review history of every article we publish publicly available.

When an article is published we post the peer reviewers' comments and the authors' responses online. We also post the versions of the paper that were used during peer review. These are the versions that the peer review comments apply to.

The versions of the paper that follow are the versions that were submitted during the peer review process. They are not the versions of record or the final published versions. They should not be cited or distributed as the published version of this manuscript.

BMJ Open is an open access journal and the full, final, typeset and author-corrected version of record of the manuscript is available on our site with no access controls, subscription charges or pay-per-view fees (http://bmjopen.bmj.com).

If you have any questions on BMJ Open's open peer review process please email editorial.bmjopen@bmj.com

BMJ Open

Interpreting the UK physical activity guidelines using a nationally representative sample of Scottish 10-11 year old children: different approaches create different outcomes

Journal:	*BMJ Open*
Manuscript ID	bmjopen-2017-018369
Article Type:	Research
Date Submitted by the Author:	27-Jun-2017

Complete List of Authors:	McCrorie, Paul; University of Glasgow, MRC/CSO Social & Public Health Sciences Unit<br>Mitchell, Richard; University of Glasgow, MRC/CSO Social & Public Health Sciences Unit<br>Ellaway, Anne; University of Glasgow, MRC/CSO SPHSU

**Primary Subject Heading**:	Public health
Secondary Subject Heading:	Sports and exercise medicine, Public health
Keywords:	Exercise, Child, Prevalence

SCHOLARONE™ Manuscripts

For peer review only

Interpreting the UK physical activity guidelines using a nationally representative sample of Scottish 10-11 year old children: different approaches create different outcomes

Paul McCrorie, Rich Mitchell, Anne Ellaway

Corresponding author contact

Dr Paul McCrorie
MRC/CSO Social and Public Health Sciences Unit
University of Glasgow
200 Renfield street
Glasgow
G2 3QB
paul.mccrorie@glasgow.ac.uk
0141 353 7620

Institutions

Paul McCrorie – see below for institution
Richard Mitchell
Anne Ellaway

MRC/CSO Social and Public Health Sciences Unit
University of Glasgow
Glasgow
Scotland

Keywords

Exercise
Child
Prevalence

Word count

3,683 exc abstract

For peer review only

Abstract

Objectives: To describe the objectively measured levels of physical activity (PA), sedentary time, and adherence to the UK PA guidelines among a nationally representative 10-11 year old cohort across Scotland, and identify the discrepancy in PA prevalence estimates conditional on the analytical decisions made.

Design: Nationally representative longitudinal cohort study.

Setting: Scotland wide in partnership with the Growing up in Scotland (GUS) longitudinal birth cohort study. Data collection took place between May 2015 and May 2016.

Participants: The parents of 2,402 GUS children were approached, and 2,162 consented to contact for the study. 1,096 children wore accelerometers for 8 consecutive days and 774 children (427 girls, 357 boys) met inclusion criteria.

Primary and secondary outcome measures: Total PA (cpm); time spent sedentary and in moderate-to-vigorous PA (MVPA); proportion of children with ≥60 mins MVPA on each day of wear (*daily approach*); proportion of children with ≥60mins of MVPA on average across days of wear (*average approach*) - presented across boys and girls, index of multiple deprivation, and season.

Results: Mean PA level was 648cpm (95% CI, 627-670). Children spent 7.5 hours (7.4-7.6) sedentary/day and 72.6 mins (70.0 -75.3) in MVPA/day. 11% (daily) and 68% (average) of children achieved the recommended levels of PA; a greater proportion of boys met the guidelines (12.5% vs. 9.8%, n.s; 75.9% vs. 59.5%, $p<0.001$); guideline prevalence exhibited seasonal variation. No significant socio-economic patterning existed across any outcome measure.

For peer review only

For peer review only - http://bmjopen.bmj.com/site/about/guidelines.xhtml

Conclusions: Inconsistencies exist regarding the analytical approach used to assess PA prevalence, resulting in markedly dissimilar outcomes. Approach choice will have a substantial impact on the development and evaluation of interventions and policy objectives. Our knowledge regarding the health implications of differing PA levels/patterns needs to advance to ensure public health messages evolve if required, but in the meantime both methods should be promoted in publications.

Article Summary

Strengths and Limitations of this study

- This is the first large-scale nationally representative physical activity study in 10-11 year old children across Scotland.
- The study demonstrates the substantial impact of alternative analytical methods on the prevalence of physical activity levels.
- Study results are comparable to other UK based accelerometry studies, which provides confidence in published physical activity levels in similar age groups.
- Non-waterproof waist-mounted accelerometers have known limitations such as their inability to measure water-based activity and to record the acceleration of upper body movement.

For peer review only

Introduction

The benefits of an active lifestyle in children and young people are well established, and include the management of overweight and obesity, improved musculoskeletal health, and a number of cardiovascular/metabolic benefits (e.g. lower blood pressure, cholesterol and blood lipids level) [1]. In 2011, a UK wide consensus among the four Chief Medical Officers (CMOs) advocated that young people under the age of 16 should accumulate at least 60 minutes, and up to several hours, of moderate to vigorous intensity physical activity (MVPA) every day [2] and, for the first time, recommended that young people should minimise time spent being sedentary.

National and international evidence on the prevalence of physical activity comes from a combination of both self-reported (e.g. survey/questionnaire/interview) and objective (e.g. accelerometry) measurements, with results often discrepant. Among Scottish children for instance, the 2015 Scottish Health Survey (SHeS) suggested that 86% of boys and 79% of girls aged between 8-10 years old meet the current CMO guidelines [3]; however, these figures have been challenged [4] as disproportionately high. Previous UK/European-based objectively measured physical activity studies have suggested that less than 10% of children and adolescents meet the recommended level of MVPA [e.g.5, 6], with some variability across European countries [7]. Self-reported measurements are often criticised for the potential introduction of recall bias, social desirability concerns, and general misunderstanding of questions [8]. However, the arguably more robust, objectively measured prevalence estimates also produce equivocal guideline adherence estimates. Yet studies using accelerometry also face methodological challenges. Previous studies

For peer review only - http://bmjopen.bmj.com/site/about/guidelines.xhtml

For peer review only

have identified issues regarding epoch length, ‘cut off’ points/thresholds used to classify MVPA, and differing measurement devices and models [4, 9], all of which have the potential to influence the underlying construct of MVPA.

A further concern which transcends both self-reported and objective measurement methods is the approach used to classify participants as either meeting, or not meeting the physical activity guidelines, and the effect of this on the resulting prevalence rates. Current UK guidelines suggest that children must meet the threshold of 60 minutes of MVPA *every day* to be considered ‘compliant’. However, some national level surveys, such as the SHeS, assess whether 60 minutes of MVPA per day is achieved *on average* across 7 days in order to assess prevalence of meeting the guidelines. The UK wide Millennium Cohort Study (MCS), for example found that 53% of 7/8 year old children met the guidelines using the average approach [10], whereas, the self-reported Health Behaviour in School aged Children (HSBC) and the parental-proxy reported Health Survey for England (HSE) found 25% (11 years old) and 21% (8-10 years old) of children met the current recommended guidelines using the more stringent ‘every day’ approach [11]. These are substantial differences that could have serious implications, not least for the evaluation and development of policy. As far as we are aware, only one study has investigated this issue with objectively measured data [12]. In an Estonian sample of 7-13 year olds (n=472), the authors demonstrated a more than twofold increase in prevalence estimates between an ‘every day’ guideline approach compared to an average approach (24% vs. 52%), although adherence to the daily approach was based on

For peer review only

meeting the guidelines on 4 out 5 weekdays (where no weekend days were included).

An additional issue with regard to guideline adherence is the 60-minute threshold. Current evidence suggests that meeting the guidelines has a positive health impact [13], yet no evidence exists to suggest that this is the definitive threshold at which benefits are gained or lost. In fact, evidence generally suggests that some activity is better than no activity, but ‘more is better’ [14]. Little evidence exists demonstrating the impact on adherence prevalence rates of altering this threshold by even small margins, and more knowledge on this may be of wider value to behaviour change interventions [12].

Given this apparent inconsistency in the assessment of children’s physical activity guideline adherence, we compared the proportion of Scottish children currently meeting the current CMO defined physical activity guidelines when (i) a *minimum* of 60 minutes of MVPA on each day of valid data was required to be classified as adherent (which we term the *daily* approach); and ii) the *mean* MVPA across valid days was ≥60 minutes in total (which we term the *average* approach). Additionally, we investigated the impact of altering the 60 minute threshold on prevalence rates for both approaches (daily and average), comparing 50, 55, 65, and 70 minutes. We also describe levels of, and gender/ socio-economic/seasonal differences in, physical activity levels in Scottish children.

For peer review only

Methods

To examine these questions we drew on the SPACES (Studying Physical Activity in Children's Environments across Scotland) study, the aim of which was to explore the environmental determinants of physical activity by conducting a large scale, nationally representative, accelerometry and Global Positioning Systems (GPS) observational study. The participants involved in SPACES were recruited from the Growing up in Scotland (GUS) study, a nationally representative longitudinal cohort study originating in 2005. As part of the Sweep 8 interviews (conducted between September 2014 and February 2015 when the children were aged approximately 10 years old), parents and children were provided with brief information about the SPACES study and asked if their contact details could be passed on to SPACES staff. From a possible 2,402 children who had participated in GUS sweep 8 interviews, 90% (n=2,162) of parents consented to be contacted by us, and we sent study information, registration documents, and consent forms by post using the main parent/carer as primary contact. The data collection for SPACES took place between May 2015 and May 2016 and ethical approval was provided by the College of Social Sciences, University of Glasgow (CSS ref: 400140067).

From the 2,162 GUS children who consented to be contacted, 1,096 (51%) children took part in the data collection. From these 1,096 participants, 774 children (417 girls; 357 boys) provided at least 4 weekdays of data and at least 1 day of weekend data. Data from the supplementary file compares our weighted sample with that of the GUS weighted Sweep 8 sample to infer representativeness (the GUS weighted sample being considered to represent the population from which it came). In

For peer review only - http://bmjopen.bmj.com/site/about/guidelines.xhtml

general, the weighting was successful across all variables. Compared to the entire GUS sweep 8 sample, our weighted sample slightly under-represented those in lowest and highest income bands (<£3,999 – £9,999; >50k), those whose parents were married, those whose mothers were aged under 20 years old at the birth of their child, those whose parents had lower level educational grades or equivalent, and those who reside in urban areas of Scotland. The sample slightly over-represented those who were cohabiting or single, and in the highest income band (>£50k).

Measurement

To assess the frequency, intensity and duration of physical activity, participants who consented to participate in the data collection were provided with a validated [15, 16] accelerometer (ActiGraph GT3X+) and asked to wear the device over 8 consecutive days; 10 hours on weekdays, and 8 hours on weekend days [17]. Children were asked to remove the accelerometer when bathing or during other water-based activities, or during contact sports or activities. Following the measurement protocol set out by the International Physical Activity and Environment Network (IPEN), we identified that a device was not being worn if there were 60 consecutive minutes of zero acceleration recorded by the device and these periods were removed from any analyses. From the same recommendations, children who provided at least 5 days including 4 weekdays and 1 weekend day were included in the analyses [18].

Design

For peer review only - http://bmjopen.bmj.com/site/about/guidelines.xhtml

Physical activity analysis

Proprietary software from the accelerometer manufacturer (ActiLife v6.11.9) was set to save PA data in 10-second epochs; PA information was digitised and stored as 'counts' – a unitless, representation of acceleration for that period.

The primary 'overall' physical activity measure was the participant's counts per minute (cpm) - a measure of total physical activity that integrates all movement recorded through the device over the duration of the device-wearing period (total counts divided by total wear time). These 'counts' were used to calculate time spent sedentary and in each intensity of physical activity by using an evidence-based threshold classification [19, 20]: sedentary (<100 cpm); light (101-2295cpm); and moderate to vigorous (>2296cpm). The proportion of children meeting the physical activity guidelines was classified through two different approaches:

Firstly, in strict agreement with the CMO statement [2], we calculated the proportion who recorded at least 60 minutes of MVPA *on each valid day of recording (*the *daily* approach*)*.

Secondly, we calculated the proportion of children who achieved an *average* of at least 60 minutes of MVPA across valid days of recording (the *average* approach*)*.

To explore the impact of varying the 60-minute threshold we then recalculated these proportions using alternative thresholds (50, 55, 65, and 70 minutes).

Prevalence estimates

Prevalence estimates were calculated for boys and girls and across a measure of multiple area-level deprivation using ranked scores (grouped into quintiles) from the Scottish Index of Multiple Deprivation (SIMD) [21]. This is a well-validated measure

For peer review only - http://bmjopen.bmj.com/site/about/guidelines.xhtml

which captures the relative social, economic, environmental and health circumstances of local populations. Seasons were classified into a four-level categorical variable based on the astronomical calendar.

Statistical Analysis

Analyses were conducted using STATA v.13 (STATA Corporation, Texas, USA). Sampling weights were used to account for the clustered and stratified sample design of the GUS cohort [22]. Weights were applied to allow for non-consent to contact, and non-consent and non-compliance of those invited to take part. Multiple linear regression models, allowing for the survey's design, were conducted on continuous outcome variables (Light, MVPA, and Sedentary time) controlling for number of valid days, mean wear time per day, and season of measurement. Logistic regression was used to model the proportion of children meeting the physical activity guidelines. All variables within the multiple linear regression analyses were included in the logistic regression models and included interaction terms for season and sex. Models were run separately for sex and SIMD. All pairwise and post-estimation tests were Bonferroni corrected to adjust for multiple comparisons.

Results

Adjusted means of derived sedentary and physical activity outcomes are summarised in Table 1. Mean physical activity levels were 648cpm, with boys exhibiting higher levels of total activity than girls (this difference just failed to reach significance at the 5% level (p=0.06). On average, children spent 7.5 hours sedentary

For peer review only - http://bmjopen.bmj.com/site/about/guidelines.xhtml

per day, with no differences between boys and girls. On average, children spent 73 mins per day in MVPA, where boys exhibited significantly higher levels of MVPA than girls (77mins vs 68 mins p<0.001). There was no significant socio-economic patterning across any outcome variable, although those in the most deprived quintile did seem to exhibit higher total activity, MVPA, % meeting the *daily* approach guidelines, and lower sedentary time than other quintiles of multiple deprivation.

Table 1 Physical activity and sedentary outcomes (N=774)

	N (weighted)	Total PA (cpm)	Sedentary All days (hrs)	Light All days (hrs)	MVPA All days (mins)	Daily Guideline prevalence	Average Guideline prevalence
**All Children**	774	648	7.5	4.2	72.6	11.1%	68.3%
**Mean (95% CI)**		(627 - 670)	(7.4 - 7.6)	(4.2 - 4.3)	(70.0 - 75.3)	(7.4 – 14.7)	(63.3 – 73.3)
**Boys**	359	663 (p = 0.06)	7.5	4.2	**77.5**	12.5%	75.9%
		(638 - 688)	(7.3 - 7.6)	(4.1 - 4.3)	(74.1 – 80.9)*	(6.7 – 18.2)	(70.4 – 81.5)
**Girls**	415	634	7.6	4.3	**67.7**	9.8%	59.5%
		(606 - 661)	(7.4 - 7.7)	(4.2 - 4.4)	(64.4 – 71.0)	(5.6 – 14.0)	(63.3 – 73.3)
**SIMD Quintile‡**							
**Most Deprived**	164	668	7.3	4.4	75.0	19.2%	68.4%
		(607 - 728)	(7.0 - 7.7)	(4.2 - 4.6)	(67.0 - 83.0)	(8.4 – 30.1)	(56.0 – 80.8)
**Quintile 2**	140	631	7.7	4.1	70.7	8.6%	69.3%
		(577 - 685)	(7.4 – 7.9)	(3.9 - 4.3)	(64.7 - 76.8)	(0.4 – 16.8)	(56.6 – 82.0)
**Quintile 3**	143	656	7.5	4.3	72.8	9.5%	65.8%
		(625 - 688)	(7.3 - 7.6)	(4.2 - 4.4)	(68.0 – 77.5)	(4.0 – 15.0)	(56.7 – 74.9)
**Quintile 4**	166	645	7.5	4.3	71.0	9.8%	66.2%
		(607 - 683)	(7.4 - 7.7)	(4.2 - 4.4)	(66.5 – 75.5)	(3.0 – 16.6)	(55.8 – 76.5)
**Least Deprived**	161	635	7.6	4.1	71.4	7.1%	65.8%
		(626 - 668)	(7.5 - 7.7)	(4.2 - 4.3)	(68.2 – 74.7)	(3.4 – 10.7)	(58.2 – 73.4)

Statistically significant difference between boys and girls, or across SIMD quintiles: *p<0.001
Figures are estimated marginal means or adjusted predicted probabilities and significance testing adjusted for season of measurement, mean wear time, and number of valid days.
‡Significance testing conducted as contrast between base category (most deprived) and all other quintiles

For peer review only - http://bmjopen.bmj.com/site/about/guidelines.xhtml

Prevalence estimates – Impact of changing analytical approach and adherence criteria

Using the *daily* definition of adherence to assess guideline prevalence, approximately 11% of all children met the current recommendations. There was no significant difference between boys (12.5%) and girls (9.8 %). No statistically significant patterns were found by area deprivation, although a slightly higher proportion of those in the most deprived quintile met the guidelines (19.2%, see Table 1). Although more boys met the guideline across all seasons except the autumn, there was no significant interaction (p=0.7) between gender and season (Figure 1).

Using the *average* approach, 68% of children achieved the recommended level of activity, and significantly more boys met the guidelines than girls (76.0% vs. 59.5%). No significant differences were evident between quintiles of deprivation. Contrary to the daily approach, there was a significant interaction between gender and season (p=0.047): a significantly higher proportion of boys met the 60-minute daily average in winter and autumn. However, a higher non-significant proportion of girls met the 60-minute daily average in summer compared to boys (see Figure 1).

Figure 2 illustrates the impact of varying the physical activity guideline reference point around 60 minutes. For both approaches, as expected, there was a linear increase in the proportion of children meeting the guidelines as the reference point was lowered from 70 minutes to 50 minutes. No significant differences existed between boys and girls at any reference point for the daily approach, although we observed a widening of the gap between them as the reference point was lowered.

For peer review only

For peer review only - http://bmjopen.bmj.com/site/about/guidelines.xhtml

Conversely, there was a consistent significant difference between boys and girls across all levels when using the average approach, albeit with a tendency for the gap to reduce as the reference point was lowered.

INSERT FIGURE 1 HERE

INSERT FIGURE 2 HERE

Discussion

Statement of Principal Findings

This was the first representative study of objectively measured physical activity and sedentary levels among 10-11 year old children across Scotland. Using the daily measure (which required children to have at least 60 minutes of MVPA on every valid day of recording), only 11% of Scottish 10-11 year olds met the recommended guideline. However, this figure substantially increased to 68% when implementing the average measure (which required children to have at least 60 minutes daily MVPA on average across all valid days). Our results also indicated that reducing or increasing the 60-minute threshold by even as little as five minutes can substantially impact the prevalence rates. If the threshold was reduced to 55-minutes per day, we predict an increase in between 2-8 percentage points depending on sex and type of analysis (using an average or daily approach).

For peer review only - http://bmjopen.bmj.com/site/about/guidelines.xhtml

For peer review only

Strengths and limitations

This study was the first to investigate the objectively measured physical activity levels and guideline prevalence in a large, nationally representative, sample of 10-11 year old Scottish children. The data reduction protocol followed those of other large studies [6], allowing accurate comparisons to be made between this study and others, particularly with consideration to the cut points advocated in the literature to classify sedentary behaviour (<100 cpm) and MVPA (>2296cpm) [6]. A further strength of this study is the comparison of two approaches to the interpretation of guideline adherence, and assessment of the subsequent impact on interpretation of physical activity levels.

The study also had limitations. Waist mounted devices are typically poor at recording the acceleration associated with cycling or upper body dominant activities. The devices were removed for water-based activities and contact sports so we will have underestimated these activities. Additionally, whilst our chosen cut points to classify MVPA were evidence based [19], other published cut points are available and their use would significantly alter our results.

Comparison with literature

The PA levels from this study are largely comparable to other large-scale, UK, objectively measured studies of PA in similar age groups (See Table 2).

For peer review only - http://bmjopen.bmj.com/site/about/guidelines.xhtml

Table 2 - Descriptive data from large, UK based, accelerometry studies using similar age groups

Study name	Setting	Year	Mean age (years)	Sample size	Mean CPM	Cut point for Sed/MVPA	Sedentary (hours)	MVPA (mins)	Prevalence of PA (%)
**SPACES**	Scotland wide	2015-2016	11.1	774	622	<100 >2296	7.5	72	11/68.3%
**MCS**	UK (Scotland Sample)	2008-2009	7.5	761	615	<100 >2241	6.4	62	52.5%
**GMS**	North East England	2008-2009	9.3	405	643	<1100 >3200	Not reported	24	5.7%
**SPEEDY**	South East England	2007	10.2	1,862	665	<100 >2000	7.6	74	70.5%
**ALSPAC**	South West England	2003-2005	11.8	5,595	580	<100 >3600	7.2	20	2.5%

SPACES: Studying Physical Activity in Children's Environments across Scotland
SPEEDY: Sport, Physical activity and Eating behaviour: Environmental Determinants in Young people [34]
ALSPAC: Avon Longitudinal Study of Parents and Children [5]
MCS: Millennium Cohort Study [10]
GMS: Gateshead Millennium Cohort Study [35]

The literature is relatively consistent in showing differences in physical activity levels between boys and girls and the recent publication by Cooper and colleagues [6], with over 6,000 9-10 year olds across 7 different countries, supports this. We did not find any statistically significant socioeconomic gradient in activity levels, although there was a tendency for those in the most deprived areas to engage in higher levels of total activity, MVPA, and less time sedentary than other quintiles of deprivation. Again, recent work from the Millennium Cohort study [10], and previous other large scale objective measured studies [5, 23] have shown little socioeconomic patterning at this age. It has been suggested, however, that active travel behaviours may be more prevalent among children from poorer neighbourhoods. They may, for example, be more likely to walk or take public

For peer review only - http://bmjopen.bmj.com/site/about/guidelines.xhtml

transport to school compared to children from more affluent neighbourhoods who are more likely to be driven to school [24]. This could explain the slightly higher levels of MVPA among children in more deprived neighbourhoods in our study. Future work with the SPACES dataset will explore this question. The seasonal variation evident in the present study was also consistent with previous work in the UK [25, 26]. Atkin and colleagues [27] for instance, found similar patterns across season but with boys consistently more active than girls when exploring time spent in MVPA. From an intervention, behaviour change, and policy perspective, it is vitally important to tailor our approaches to consider the differential effects of season on PA levels, particularly for girls. More importantly, future work should continue to investigate the determining factors associated with seasonal variation, particularly the potential moderating effects produced by the natural and built environment on PA behaviours during winter and autumn compared to spring and summer. This is another important question that the SPACES study will explore using collected GPS data and Geographic Information Systems (GIS) software.

Our most important finding is that either 11% or 68% of Scottish children meet the current UK guidelines, depending on the approach taken to measurement. This difference could be interpreted as either 'quite poor' or 'relatively good' and would have serious implications no matter which interpretation was taken. The only other study, known to the authors, who have published data using similar approaches, was conducted in Estonian children and young people (6-13 years old). Mooses and colleagues [12], using an identical MVPA 'cut point' (i.e. the Evenson cut point), identified a greater than two-fold increase in guideline prevalence dependent on

For peer review only - http://bmjopen.bmj.com/site/about/guidelines.xhtml

approach (daily, 24% and average, 52%). However, the authors did not include weekend days, and daily adherence was classified as meeting 4 out 5 weekdays. The present study required a minimum of 4 weekdays and 1 weekend day to be included in analyses, and adherence to the daily approach was based on meeting each and every valid day of wear. Furthermore, the present study was specific to 10/11 year olds and nationally representative. Therefore, our results aren't directly comparable. Previous studies have also identified the impact on prevalence estimates when using different 'cut points' to discern MVPA. The results from work by Griffiths and colleagues [10], indicated that the proportions of children meeting the PA guidelines changed from 84.0%/59.4% (boys and girls respectively) to 13.7%/0.4%, when altering their cut point from 2000cpm to 3000cpm. There have been calls for an international consensus process on accelerometry methodology and reporting standards in youth, with a formal review of recommendations every 5 years to evaluate new science [18]. The recent creation of the International Children's Accelerometry Database [ICAD, 6] where standardised methods have re-analysed and re-integrated data from multiple international studies leading to cross-country comparisons is an important step in recognising the need to standardise measurement approaches.

The question remains however, which approach is more appropriate. Does activity 'every day' matter more, in health benefit terms, than the accumulation over the week? Is 'missing' one day out of one week problematic in terms of health [1, 28]? Furthermore, although the latest evidence demonstrates that meeting the 60 minutes of MVPA per day has positive health implications, particularly for adiposity

For peer review only

and quality of life [13], there is little evidence on whether individuals whose activity levels approach, but do not reach, 60 minutes per day are less healthy, and whether narrowly falling short of ‘every day’ has implications for health outcomes [12]. We know that activity under the prescribed intensity of MVPA has substantial benefits on the maintenance of energy balance, i.e. Non-Exercise Activity Thermogenesis (NEAT) [29, 30]; evidence is growing of the benefits of light intensity activity on other aspects of health, such as the reduced risk of all-cause mortality [31], and the inverse association with type-2-diabetes risk factors (e.g. 2-h plasma glucose levels) in adults [32]. As far as the authors are aware, no literature exists on the optimal levels, and patterns, of physical activity intensities combined. Our results also highlight that the proportions of children meeting the UK guidelines vary considerably across 10 minutes either side of the 60-minute threshold. It would be beneficial if studies elsewhere could investigate the health effects of differing patterns of daily MVPA (and sedentary and light PA) and the health related implications of changing the 60-minute threshold. Doing so may result in greater flexibility around guideline adherence, and will provide researchers with greater knowledge when communicating health messages to the public.

Implications for Policy

In 2014, Scotland launched the Active Scotland Outcomes Framework (ASOF), which set out ambitions for physical activity and sport in Scotland over the next 10 years. A range of indicators will track progress using SHeS data. Alongside the framework is a key national legacy 10 year programme designed to influence population levels of activity in adults and children [33]. The extent to which these initiatives are viewed

For peer review only - http://bmjopen.bmj.com/site/about/guidelines.xhtml

For peer review only

as successful in increasing children and young people's physical activity will depend on the measures used to assess physical activity and we hope that our findings can inform the understanding of physical activity prevalence, as well as evaluation methods used to measure the impact of policy change. Griffiths and colleagues [10] were right to state that the clarity of cut points used for MVPA is vital for monitoring of policy objectives around physical activity but we also demonstrate here that the approach used to assess guideline adherence will determine the result and, therefore, the evaluation of interventions and policy objectives. It is our position that both the *daily* and *average* approach be presented in all future surveillance papers and governmental publications. This is a significant policy issue. Researchers should work with policy makers to communicate the intricacies of physical activity prevalence interpretation.

Acknowledgements

We would like to thank the children from the Growing up in Scotland longitudinal birth cohort study for taking part in the research and to members of the Scotcen Social Research team who assisted along the way.

Competing Interests

The authors declare no competing interests

Funding

This work was supported by the Medical Research Council [grant number MC_UU_12017/10]; and the Scottish Government [grant number SR/SC 17/04/2012].

For peer review only - http://bmjopen.bmj.com/site/about/guidelines.xhtml

Author contribution statement

PM and AE ran the data collection process, and PM led on data processing and data analysis and drafted the article. RM and AE assisted with statistical analysis and interpretation of the data, and RM contributed to the graphical presentation of the results. AE and RM critically revised the article and all authors signed off the final draft.

Data Sharing Statement

We are committed to maximizing the use of SPACES study data to advance knowledge to improve young people's health, and welcome proposals for collaborative projects and data sharing. Our data sharing policy follows that of the Medical Research Council and aims to balance making data as widely and freely available as possible with safeguarding the privacy of participants, protecting confidential data, and maintaining the reputation of the study. Please contact Prof Anne Ellaway (anne.ellaway@glasgow.ac.uk) for further information.

For peer review only

References

1. Janssen, I. and A.G. Leblanc, *Systematic review of the health benefits of physical activity and fitness in school-aged children and youth.* Int J Behav Nutr Phys Act, 2010. **7**: p. 40.
2. Department of Health, *Start Active, Stay Active: a report on physical activity from the four home countries' Chief Medical Officers*, D.o. Health, Editor. 2011, Crown Copyright: London.
3. Hovald, P., *Chapter 7 Physical activity*, in *The Scottish Health Survey 2015 edition: volume1, main report. A National Statistics Publication for Scotland*, D. Campbell-Jack, S. Hinchliffe, and L. Rutherford, Editors. 2016, Scottish Government: Edinburgh.
4. Basterfield, L., et al., *Surveillance of physical activity in the UK is flawed: validation of the Health Survey for England Physical Activity Questionnaire.* Arch Dis Child, 2008. **93**(12): p. 1054-8.
5. Riddoch, C.J., et al., *Objective measurement of levels and patterns of physical activity.* Arch Dis Child, 2007. **92**(11): p. 963-9.
6. Cooper, A.R., et al., *Objectively measured physical activity and sedentary time in youth: the International children's accelerometry database (ICAD).* Int J Behav Nutr Phys Act, 2015. **12**: p. 113.
7. Konstabel, K., et al., *Objectively measured physical activity in European children: the IDEFICS study.* Int J Obes (Lond), 2014. **38 Suppl 2**: p. S135-43.
8. Welk, G.J., C.B. Corbin, and D. Dale, *Measurement issues in the assessment of physical activity in children.* Res Q Exerc Sport, 2000. **71**(2 Suppl): p. S59-73.
9. Corder, K., et al., *Assessment of physical activity in youth.* J Appl Physiol (1985), 2008. **105**(3): p. 977-87.
10. Griffiths, L.J., et al., *How active are our children? Findings from the Millennium Cohort Study.* BMJ Open, 2013. **3**(8): p. e002893.
11. World Health Organization, W., *Health Behaviour in School-Aged Children (HBSC) Study: International Report from the 2013/2014 Survey*, W.-. Europe, Editor. 2016, World Health Organisation: Copenhagen, Denmark.
12. Mooses, K., et al., *Different Methods Yielded Two-Fold Difference in Compliance with Physical Activity Guidelines on School Days.* PLoS One, 2016. **11**(3): p. e0152323.
13. Poitras, V.J., et al., *Systematic review of the relationships between objectively measured physical activity and health indicators in school-aged children and youth.* Appl Physiol Nutr Metab, 2016. **41**(6 Suppl 3): p. S197-239.
14. Tremblay, M.S., et al., *New Canadian physical activity guidelines.* Appl Physiol Nutr Metab, 2011. **36**(1): p. 36-46; 47-58.
15. Robusto, K.M. and S.G. Trost, *Comparison of three generations of ActiGraph activity monitors in children and adolescents.* J Sports Sci, 2012. **30**(13): p. 1429-35.
16. Romanzini, M., et al., *Calibration of ActiGraph GT3X, Actical and RT3 accelerometers in adolescents.* Eur J Sport Sci, 2014. **14**(1): p. 91-9.

For peer review only - http://bmjopen.bmj.com/site/about/guidelines.xhtml

17. Rowlands, A.V., E.L. Pilgrim, and R.G. Eston, *Patterns of habitual activity across weekdays and weekend days in 9-11-year-old children.* Prev Med, 2008. **46**(4): p. 317-24.
18. Cain, K.L., et al., *Using accelerometers in youth physical activity studies: a review of methods.* J Phys Act Health, 2013. **10**(3): p. 437-50.
19. Evenson, K.R., et al., *Calibration of two objective measures of physical activity for children.* J Sports Sci, 2008. **26**(14): p. 1557-65.
20. Trost, S.G., et al., *Comparison of accelerometer cut points for predicting activity intensity in youth.* Med Sci Sports Exerc, 2011. **43**(7): p. 1360-8.
21. Government, S., *Scottish Index of Multiple Deprivation: SIMD16 Technical Notes.* 2016, National Statistics.
22. Bradshaw, P., et al., *Growing Up In Scotland Sweep 1 - 2005 User Guide*. 2005, Scottish Centre for Social Research: Edinburgh.
23. van Sluijs, E.M., et al., *Physical activity and dietary behaviour in a population-based sample of British 10-year old children: the SPEEDY study (Sport, Physical activity and Eating behaviour: environmental Determinants in Young people).* BMC Public Health, 2008. **8**: p. 388.
24. GCPH, *Children's travel to school – are we moving in the right direction*, G.C.f.P. Health, Editor. 2011, GCPH: Glasgow.
25. Rich, C., L.J. Griffiths, and C. Dezateux, *Seasonal variation in accelerometer-determined sedentary behaviour and physical activity in children: a review.* Int J Behav Nutr Phys Act, 2012. **9**: p. 49.
26. Atkin, A.J., et al., *Seasonal Variation in Children's Physical Activity and Sedentary Time.* Med Sci Sports Exerc, 2016. **48**(3): p. 449-56.
27. Atkin, A.J., et al., *Seasonal Variation in Children's Physical Activity and Sedentary Time.* Medicine and Science in Sports and Exercise, 2016. **48**(3): p. 449-456.
28. Bull, F. and E.W. Group, *Physical Activity Guidelines in the U.K.: Review and Recommendations.* 2010, Loughborough University: School of Sport, Exercise and Health Sciences, Loughborough University.
29. Levine, J.A., *Nonexercise activity thermogenesis--liberating the life-force.* J Intern Med, 2007. **262**(3): p. 273-87.
30. Katzmarzyk, P.T., *Physical activity, sedentary behavior, and health: paradigm paralysis or paradigm shift?* Diabetes, 2010. **59**(11): p. 2717-25.
31. Loprinzi, P.D., *Light-Intensity Physical Activity and All-Cause Mortality.* Am J Health Promot, 2016.
32. Healy, G.N., et al., *Objectively measured light-intensity physical activity is independently associated with 2-h plasma glucose.* Diabetes Care, 2007. **30**(6): p. 1384-9.
33. Scottish Government, *Physical Activity Implementation Plan: A More Active Scotland*. 2014, Scottish Government.
34. Steele, R.M., et al., *Targeting sedentary time or moderate- and vigorous-intensity activity: independent relations with adiposity in a population-based sample of 10-y-old British children.* Am J Clin Nutr, 2009. **90**(5): p. 1185-92.
35. Basterfield, L., et al., *Longitudinal study of physical activity and sedentary behavior in children.* Pediatrics, 2011. **127**(1): p. e24-30.

For peer review only - http://bmjopen.bmj.com/site/about/guidelines.xhtml

For peer review only - http://bmjopen.bmj.com/site/about/guidelines.xhtml

Supplementary table comparison of weighted sample to known national level SES/demographic distributions

Demographic characteristic	SPACES weighted sample distribution	Sweep 8 Growing up in Scotland (GUS) weighted sample distribution
Income (per annum)		
<3,999 - £9,999	3%	5%
£10,000 - £19,999	20%	21%
£20,000 - £28,999	18%	16%
£29,000 - £37,999	14%	14%
£38,000 - £49,999	14%	15%
>50,000	27%	29%
Mothers age at birth (years)		
Under 20	4%	7%
20 -29	43%	41%
30 – 39	50%	49%
40 or older	3%	3%
Marital status		
Married	61%	68%
Cohabiting	19%	15%
Single	11%	9%
Widowed	1%	1%
Divorced	5%	3%
Separated	3%	4%
SIMD quintile (2012)		
Most	21%	20%
2nd	18%	21%
Middling	19%	20%
4th	21%	18%
Least Deprived	21%	21%
Highest educational qualification in household		
No qualification	3%	6%
Lower level Standard Grades or equivalent	3%	4%
Upper level Standard Grades or equivalent	19%	19%
Higher Grades or equivalent	39%	33%
Degree level academic or equivalent	35%	38%
Other	1%	0.4%
Urban/Rural dwelling		
Large urban	36%	38%
Other Urban	33%	32%
Small accessible towns	9%	10%
Small remote towns	3%	3%
Accessible rural	12%	13%
Remote rural	7%	4%

For peer review only

For peer review only - http://bmjopen.bmj.com/site/about/guidelines.xhtml

BMI UK categories		
Underweight	2%	2%
Healthy weight	64%	64%
Overweight	18%	15%
Obese	16%	19%

For peer review only

For peer review only - http://bmjopen.bmj.com/site/about/guidelines.xhtml

For peer review only

For peer review only - http://bmjopen.bmj.com/site/about/guidelines.xhtml

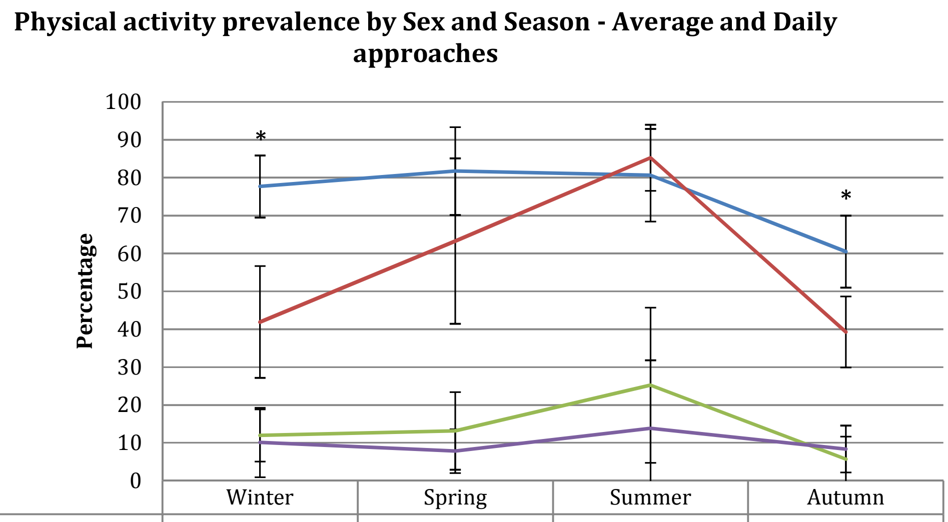

	Winter	Spring	Summer	Autumn
Male - average ‡	78	82	81	60
Female - average ‡	42	63	85	39
Male - daily	12	13	25	6
Female - daily	10	8	14	8

Figure 1- Physical activity prevalence across season of measurement by sex and measurement approach

*p<0.01; statistically significant difference between boys and girls within season

‡ Statistically significant interaction between gender and season, p<0.05

Figures are predicted probabilities and significance testing is adjusted for mean wear time, and number of valid days.

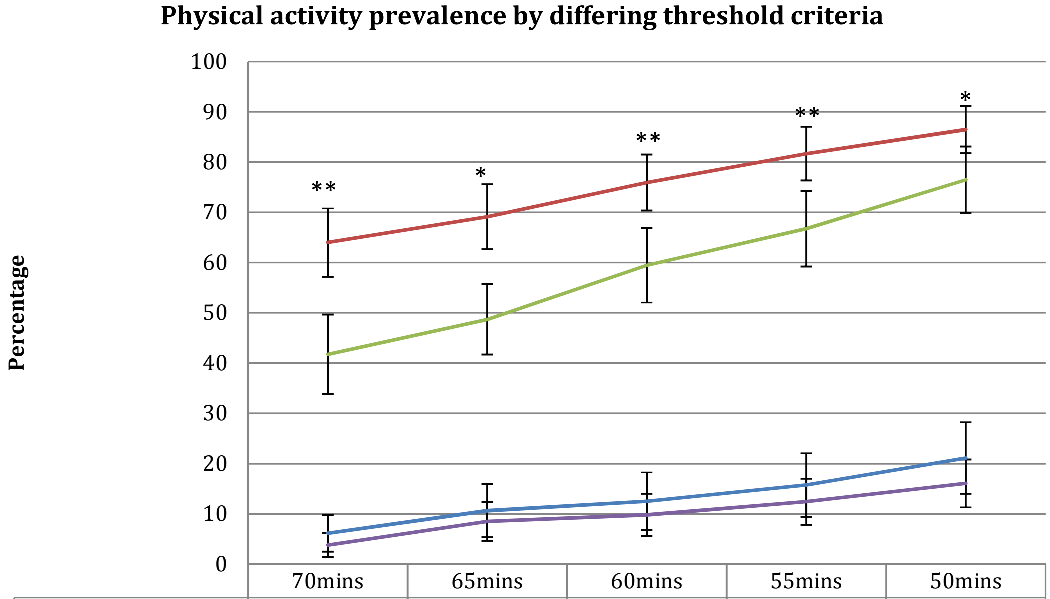

	70mins	65mins	60mins	55mins	50mins
Male - Average	64	69	76	82	86
Female - Average	42	49	59	67	77
Male - Daily	6	11	12	16	21
Female - Daily	4	9	10	12	16

Figure 1 - Physical activity prevalence by sex and threshold classification reference – daily and average approaches

*p<0.01, **p<0.001; statistically significant difference between boys and girls within threshold reference point and measurement approach

Figures are predicted probabilities and significance testing is adjusted for season of measurement, mean wear time, and number of valid days.

For peer review only - http://bmjopen.bmj.com/site/about/guidelines.xhtml

STROBE Statement—checklist of items that should be included in reports of observational studies

	Item No	Recommendation
**Title and abstract**	1	(*a*) Indicate the study's design with a commonly used term in the title or the abstract ☑ page 2
		(*b*) Provide in the abstract an informative and balanced summary of what was done and what was found ☑ page 2
**Introduction**		
Background/rationale	2	Explain the scientific background and rationale for the investigation being reported ☑ Page 4/5/6
Objectives	3	State specific objectives, including any prespecified hypotheses ☑ Page 6
**Methods**		
Study design	4	Present key elements of study design early in the paper ☑ Page 7
Setting	5	Describe the setting, locations, and relevant dates, including periods of recruitment, exposure, follow-up, and data collection ☑ Page 7/8/9
Participants	6	(*a*) *Cohort study*—Give the eligibility criteria, and the sources and methods of selection of participants. Describe methods of follow-up<br>*Case-control study*—Give the eligibility criteria, and the sources and methods of case ascertainment and control selection. Give the rationale for the choice of cases and controls<br>*Cross-sectional study*—Give the eligibility criteria, and the sources and methods of selection of participants ☑ Page7/8
		(*b*) *Cohort study*—For matched studies, give matching criteria and number of exposed and unexposed<br>*Case-control study*—For matched studies, give matching criteria and the number of controls per case
Variables	7	Clearly define all outcomes, exposures, predictors, potential confounders, and effect modifiers. Give diagnostic criteria, if applicable ☑ Page 9
Data sources/ measurement	8*	For each variable of interest, give sources of data and details of methods of assessment (measurement). Describe comparability of assessment methods if there is more than one group ☑ Page 9
Bias	9	Describe any efforts to address potential sources of bias ☑ Page 7/9
Study size	10	Explain how the study size was arrived at ☑ Page 7
Quantitative variables	11	Explain how quantitative variables were handled in the analyses. If applicable, describe which groupings were chosen and why ☑ Page 9/10
Statistical methods	12	(*a*) Describe all statistical methods, including those used to control for confounding ☑Page 10
		(*b*) Describe any methods used to examine subgroups and interactions ☑ Page 10
		(*c*) Explain how missing data were addressed
		(*d*) *Cohort study*—If applicable, explain how loss to follow-up was addressed<br>*Case-control study*—If applicable, explain how matching of cases and controls was addressed<br>*Cross-sectional study*—If applicable, describe analytical methods taking account of sampling strategy ☑ Page 10
		(*e*) Describe any sensitivity analyses

Continued on next page

For peer review only - http://bmjopen.bmj.com/site/about/guidelines.xhtml

**Results**		
Participants	13*	(a) Report numbers of individuals at each stage of study—eg numbers potentially eligible, examined for eligibility, confirmed eligible, included in the study, completing follow-up, and analysed ☑ Page 7
		(b) Give reasons for non-participation at each stage ☑ Page 7
		(c) Consider use of a flow diagram
Descriptive data	14*	(a) Give characteristics of study participants (eg demographic, clinical, social) and information on exposures and potential confounders ☑ Page 7
		(b) Indicate number of participants with missing data for each variable of interest
		(c) *Cohort study*—Summarise follow-up time (eg, average and total amount)
Outcome data	15*	*Cohort study*—Report numbers of outcome events or summary measures over time
		Case-control study—Report numbers in each exposure category, or summary measures of exposure
		Cross-sectional study—Report numbers of outcome events or summary measures ☑ Page 11
Main results	16	(*a*) Give unadjusted estimates and, if applicable, confounder-adjusted estimates and their precision (eg, 95% confidence interval). Make clear which confounders were adjusted for and why they were included ☑ Page 11/12/13
		(*b*) Report category boundaries when continuous variables were categorized
		(*c*) If relevant, consider translating estimates of relative risk into absolute risk for a meaningful time period
Other analyses	17	Report other analyses done—eg analyses of subgroups and interactions, and sensitivity analyses
**Discussion**		
Key results	18	Summarise key results with reference to study objectives ☑ Page 13
Limitations	19	Discuss limitations of the study, taking into account sources of potential bias or imprecision. Discuss both direction and magnitude of any potential bias ☑ Page 14
Interpretation	20	Give a cautious overall interpretation of results considering objectives, limitations, multiplicity of analyses, results from similar studies, and other relevant evidence ☑ Page 17/18/19
Generalisability	21	Discuss the generalisability (external validity) of the study results ☑ Page 14/15/16
**Other information**		
Funding	22	Give the source of funding and the role of the funders for the present study and, if applicable, for the original study on which the present article is based ☑ Page 19

*Give information separately for cases and controls in case-control studies and, if applicable, for exposed and unexposed groups in cohort and cross-sectional studies.

Note: An Explanation and Elaboration article discusses each checklist item and gives methodological background and published examples of transparent reporting. The STROBE checklist is best used in conjunction with this article (freely available on the Web sites of PLoS Medicine at http://www.plosmedicine.org/, Annals of Internal Medicine at http://www.annals.org/, and Epidemiology at http://www.epidem.com/). Information on the STROBE Initiative is available at www.strobe-statement.org.

For peer review only

A comparison of two methods to assess physical activity prevalence in children: an observational study using a nationally representative sample of Scottish 10-11 year old children

Journal:	*BMJ Open*
Manuscript ID	bmjopen-2017-018369.R1
Article Type:	Research
Date Submitted by the Author:	22-Sep-2017
Complete List of Authors:	McCrorie, Paul; University of Glasgow, MRC/CSO Social & Public Health Sciences Unit<br>Mitchell, Richard; University of Glasgow, MRC/CSO Social & Public Health Sciences Unit<br>Ellaway, Anne; University of Glasgow, MRC/CSO SPHSU
**Primary Subject Heading**:	Public health
Secondary Subject Heading:	Sports and exercise medicine, Public health
Keywords:	Exercise, Child, Prevalence

For peer review only

A comparison of two methods to assess physical activity prevalence in children: an observational study using a nationally representative sample of Scottish 10-11 year old children

Paul McCrorie, Rich Mitchell, Anne Ellaway

Corresponding author contact

Dr Paul McCrorie
MRC/CSO Social and Public Health Sciences Unit
University of Glasgow
200 Renfield Street
Glasgow
G2 3QB
paul.mccrorie@glasgow.ac.uk
0141 353 7620

Institutions

Paul McCrorie – see below for institution
Richard Mitchell
Anne Ellaway

MRC/CSO Social and Public Health Sciences Unit
University of Glasgow
Glasgow
Scotland

Keywords

Exercise
Child
Prevalence

Word count

3,683 exc abstract

For peer review only

For peer review only - http://bmjopen.bmj.com/site/about/guidelines.xhtml

Abstract

Objectives: To describe the objectively measured levels of physical activity (PA) and sedentary time in a nationally representative sample of 10-11 year old children, and compare adherence estimates to the UK PA guidelines using two approaches to assessing prevalence.

Design: Nationally representative longitudinal cohort study.

Setting: Scotland wide in partnership with the Growing up in Scotland (GUS) study. Data collection took place between May 2015 and May 2016.

Participants: The parents of 2,402 GUS children were approached, and 2,162 consented to contact. Consenting children (n=1,096) wore accelerometers for 8 consecutive days and 774 participants (427 girls, 357 boys) met inclusion criteria.

Primary and secondary outcome measures: Total PA (cpm); time spent sedentary and in moderate-to-vigorous PA (MVPA); proportion of children with ≥60 mins MVPA on each day of wear (*daily approach*); proportion of children with ≥60mins of MVPA on average across days of wear (*average approach*) - presented across boys and girls, index of multiple deprivation, and season.

Results: Mean PA level was 648cpm (95% CI, 627-670). Children spent 7.5 hours (7.4-7.6) sedentary/day and 72.6 mins (70.0 -75.3) in MVPA/day. Eleven percent (daily) and 68% (average) of children achieved the recommended levels of PA ($p<0.05$ for difference); a greater proportion of boys met the guidelines (12.5% vs. 9.8%, n.s; 75.9% vs. 59.5%, $p<0.001$); guideline prevalence exhibited seasonal variation. No significant socio-economic patterning existed across any outcome measure.

For peer review only - http://bmjopen.bmj.com/site/about/guidelines.xhtml

Conclusions: PA estimates are significantly influenced by the analytical method used to assess prevalence. This could have a substantial impact on the evaluation of interventions, policy objectives and public health investment. Existing guidelines which focus on daily PA only may not further our understandings about the underlying construct itself. Gender differences exist within this age-group, suggesting greater investment, with particular consideration of seasonality, is needed for targeted intervention work in younger children.

Article Summary

Strengths and Limitations of this study

- This is the first large-scale nationally representative physical activity study in 10-11 year old children across Scotland.
- The study demonstrates the substantial impact of alternative analytical methods on the prevalence of physical activity levels.
- Study results are comparable to other UK based accelerometry studies, which provides confidence in published physical activity levels in similar age groups.
- Non-waterproof waist-mounted accelerometers have known limitations such as their inability to measure water-based activity and to record the acceleration of upper body movement.

For peer review only

Introduction

The benefits of an active lifestyle in children and young people are well established, and include the management of overweight and obesity, improved musculoskeletal health, and a number of cardiovascular/metabolic benefits (e.g. lower blood pressure, cholesterol and blood lipids level) [1]. In 2011, a UK wide consensus among the four Chief Medical Officers (CMOs) advocated that young people under the age of 16 should accumulate at least 60 minutes, and up to several hours, of moderate to vigorous intensity physical activity (MVPA) every day [2] and, for the first time, recommended that young people should minimise time spent being sedentary.

National and international evidence on the prevalence of physical activity comes from a combination of both self-reported (e.g. survey/questionnaire/interview) and objective (e.g. accelerometry) measurements, with results often discrepant [3, 4]. Among Scottish children for instance, the 2015 Scottish Health Survey (SHeS) suggested that 86% of boys and 79% of girls aged between 8-10 years old meet the current CMO guidelines [3]; however, these figures have been challenged [4] as disproportionately high. Previous UK/European-based objectively measured physical activity studies have suggested that less than 10% of children and adolescents meet the recommended level of MVPA [e.g.5, 6], with some variability across European countries [7]. Self-reported measurements are often criticised for the potential introduction of recall bias, social desirability concerns, and general misunderstanding of questions [8]. However, the arguably more robust, objectively measured prevalence estimates also produce equivocal guideline adherence estimates, and studies using accelerometry also face methodological challenges. Previous studies

For peer review only

have identified issues regarding epoch length, 'cut off' points/thresholds used to classify MVPA, and differing measurement devices and models [4, 9], all of which have the potential to influence the underlying construct of MVPA.

A specific concern which transcends both self-reported and objective measurement methods is the approach used to classify participants as either meeting, or not meeting the physical activity guidelines, and the effect of this on the resulting prevalence rates. Current UK guidelines suggest that children must meet the threshold of 60 minutes of MVPA *every day* to be considered 'compliant'. However, some national level surveys, such as the SHeS, assess whether 60 minutes of MVPA per day is achieved *on average* across 7 days in order to assess prevalence of meeting the guidelines. The UK wide Millennium Cohort Study (MCS), for example found that 53% of 7/8 year old children met the guidelines using the average approach [10], whereas, the self-reported Health Behaviour in School aged Children (HSBC) and the parental-proxy reported Health Survey for England (HSE) found 25% (11 years old) and 21% (8-10 years old) of children met the current recommended guidelines using the more stringent 'every day' approach [11]. These are substantial differences that could have serious implications, not least for the evaluation and development of policy. As far as we are aware, only one study has investigated this issue with objectively measured data [12]. In an Estonian sample of 7-13 year olds (n=472), the authors demonstrated a more than twofold increase in prevalence estimates between an 'every day' guideline approach compared to an average approach (24% vs. 52%), although adherence to the daily approach was based on

For peer review only - http://bmjopen.bmj.com/site/about/guidelines.xhtml

meeting the guidelines on 4 out 5 weekdays (where no weekend days were included).

An additional issue with regard to guideline adherence is the 60-minute threshold. Current evidence suggests that meeting the guidelines has a positive health impact [13], yet no evidence exists to suggest that this is the definitive threshold at which benefits are gained or lost. In fact, evidence generally suggests that some activity is better than no activity, but 'more is better' [14]. Little evidence exists demonstrating the impact on adherence prevalence rates of altering this threshold by even small margins, and more knowledge regarding this may be of wider value to behaviour change interventions [12].

Given this apparent inconsistency in the assessment of children's physical activity guideline adherence, we compared the proportion of Scottish children currently meeting the current CMO defined physical activity guidelines when (i) a *minimum* of 60 minutes of MVPA on each day of valid data was required to be classified as adherent (which we term the *daily* approach); and ii) the *mean* MVPA across valid days was ≥60 minutes in total (which we term the *average* approach). Additionally, we investigated the impact of altering the 60 minute threshold on prevalence rates for both approaches (daily and average), comparing 50, 55, 65, and 70 minutes. We also describe levels of, and gender/socio-economic/seasonal differences in, physical activity levels in Scottish children.

For peer review only

Methods

To examine these questions we drew on the SPACES (Studying Physical Activity in Children's Environments across Scotland) study, the aim of which was to explore the environmental determinants of physical activity by conducting a large scale, nationally representative, accelerometry and Global Positioning Systems (GPS) observational study. The participants involved in SPACES were recruited from the Growing up in Scotland (GUS) study, a nationally representative longitudinal cohort study originating in 2005. As part of the Sweep 8 interviews (conducted between September 2014 and February 2015 when the children were aged approximately 10 years old), parents and children were provided with brief information about the SPACES study and asked if their contact details could be passed on to SPACES staff. From a possible 2,402 children who had participated in GUS sweep 8 interviews, 90% (n=2,162) of parents consented to be contacted by us, and we sent study information, registration documents, and consent forms by post using the main parent/carer as primary contact. The data collection for SPACES took place between May 2015 and May 2016 and ethical approval was provided by the College of Social Sciences, University of Glasgow (CSS ref: 400140067).

Measurement

To assess the frequency, intensity and duration of physical activity, participants who consented to participate in the data collection were provided with a validated [15, 16] accelerometer (ActiGraph GT3X+) and asked to wear the device over 8 consecutive days for the waking hours. We included days as valid if they had 10

For peer review only

hours on weekdays, and 8 hours on weekend days [17]. This was to balance having enough data to reliably represent daily activity, and recognise wear time is lower during weekend days (when children may be more likely to spend more time in bed). Children were asked to remove the accelerometer when bathing or during other water-based activities, or during contact sports or activities. Following the measurement protocol set out by the International Physical Activity and Environment Network (IPEN), we identified that a device was not being worn if there were 60 consecutive minutes of zero acceleration recorded by the device and these periods were removed from any analyses. From the same recommendations, children who provided at least 5 days including 4 weekdays and 1 weekend day were included in the analyses [18].

Design

Physical activity analysis

Proprietary software from the accelerometer manufacturer (ActiLife v6.11.9) was set to save PA data in 10-second epochs; PA information was digitised and stored as 'counts' – a unit-less representation of acceleration for that period.

The primary 'overall' physical activity measure was the participant's counts per minute (cpm) - a measure of total physical activity that integrates all movement recorded through the device over the duration of the device-wearing period (total counts divided by total wear time). These 'counts' were used to calculate time spent sedentary and in each intensity of physical activity by using an evidence-based threshold classification [19, 20]: sedentary (<100 cpm); light (101-2295cpm); and

For peer review only - http://bmjopen.bmj.com/site/about/guidelines.xhtml

moderate to vigorous (>2296cpm). The proportion of children meeting the physical activity guidelines was classified through two different approaches:

Firstly, in strict agreement with the CMO statement [2], we calculated the proportion who recorded at least 60 minutes of MVPA *on each valid day of recording* (the *daily* approach).

Secondly, we calculated the proportion of children who achieved an *average* of at least 60 minutes of MVPA across valid days of recording (the *average* approach).

To explore the impact of varying the 60-minute threshold we then recalculated these proportions using alternative thresholds (50, 55, 65, and 70 minutes). These were chosen to reflect what we considered to be slight variations, in both directions, in the duration threshold and would hopefully capture those children who narrowly missed the 60-minute threshold whilst also demonstrating the impact of requiring a further 5 or 10 minutes to meet the daily guidelines.

Prevalence estimates

Prevalence estimates were calculated for boys and girls and across a measure of multiple area-level deprivation using ranked scores (grouped into quintiles) from the Scottish Index of Multiple Deprivation (SIMD) [21]. This is a well-validated measure which captures the relative social, economic, environmental and health circumstances of local populations. Seasons were classified into a four-level categorical variable based on the astronomical calendar.

Statistical Analysis

Analyses were conducted using STATA v.13 (STATA Corporation, Texas, USA), and accounted for the clustered and stratified survey sample design of the GUS cohort

For peer review only - http://bmjopen.bmj.com/site/about/guidelines.xhtml

For peer review only

[22]. Sampling weights were applied to allow for non-consent to contact, and non-consent and non-compliance of those invited to take part. This approach maximises the accuracy of the point estimates and the standard errors from the analyses, therefore reducing the likelihood of Type 1 errors. For this particular analysis we were more concerned with controlling for the effects of clustering on standard error estimation rather than exploring it, and this is why we didn't use alternative methods such as multi-level modelling.

Multiple linear regression models, allowing for the survey's design, were conducted on continuous outcome variables (Light, MVPA, and Sedentary time) controlling for number of valid days, mean wear time per day, and season of measurement. Logistic regression was used to model the proportion of children meeting the physical activity guidelines. All covariates within the multiple linear regression analyses were included in the logistic regression models. All models were ran separately for 'sex' and 'SIMD' and the models exploring 'sex' included an interaction term with 'season of measurement'. All pairwise and post-estimation tests were Bonferroni corrected to adjust for multiple comparisons.

Results

From the 2,162 GUS children who consented to be contacted, 1,096 (51%) children took part in the data collection. From these 1,096 participants, 774 children (417 girls; 357 boys) provided at least 4 weekdays of data and at least 1 day of weekend data. Data from the supplementary file (see supplementary Table 1) compares our weighted sample with that of the GUS weighted Sweep 8 sample to infer

For peer review only - http://bmjopen.bmj.com/site/about/guidelines.xhtml

representativeness (the GUS weighted sample being considered to represent the population from which it came). In general, the weighting was successful across all variables. Compared to the entire GUS sweep 8 sample, our weighted sample slightly under-represented those in lowest and highest income bands (<£3,999 – £9,999; >50k), those whose parents were married, those whose mothers were aged under 20 years old at the birth of their child, those whose parents had lower level educational grades or equivalent, and those who reside in urban areas of Scotland. The sample slightly over-represented those who were cohabiting or single, and in the highest income band (>£50k).

Adjusted means of derived sedentary and physical activity outcomes are summarised in Table 1. Mean physical activity levels were 648cpm, with boys exhibiting higher levels of total activity than girls (this difference just failed to reach significance at the 5% level (p=0.06). On average, children spent 7.5 hours sedentary per day, with no differences between boys and girls. On average, children spent 73 mins per day in MVPA, where boys exhibited significantly higher levels of MVPA than girls (see Table 1). There was no significant socio-economic patterning across any outcome variable, although those in the most deprived quintile did seem to exhibit higher total activity, MVPA, % meeting the *daily* approach guidelines, and lower sedentary time than other quintiles of multiple deprivation.

Table 1 Physical activity and sedentary outcomes (N=774)

	N	Total PA	Sedentary	Light	MVPA	Daily	Average

For peer review only - http://bmjopen.bmj.com/site/about/guidelines.xhtml

	(weighted)	(cpm)	All days (hrs)	All days (hrs)	All days (mins)	Guideline prevalence	Guideline prevalence
**All Children**	774	648	7.5	4.2	72.6	11.1%	68.3%
**Mean (95% CI)**		(627 - 670)	(7.4 - 7.6)	(4.2 - 4.3)	(70.0 - 75.3)	(7.4 - 14.7)	(63.3 - 73.3)
**Boys**	359	663 (p = 0.06)	7.5	4.2	**77.5**	12.5%	75.9%
		(638 - 688)	(7.3 - 7.6)	(4.1 - 4.3)	(74.1 - 80.9)*	(6.7 - 18.2)	(70.4 - 81.5)
**Girls**	415	634	7.6	4.3	**67.7**	9.8%	59.5%
		(606 - 661)	(7.4 - 7.7)	(4.2 - 4.4)	(64.4 - 71.0)	(5.6 - 14.0)	(63.3 - 73.3)
**SIMD Quintile‡**							
**Most Deprived**	164	668	7.3	4.4	75.0	19.2%	68.4%
		(607 - 728)	(7.0 - 7.7)	(4.2 - 4.6)	(67.0 - 83.0)	(8.4 - 30.1)	(56.0 - 80.8)
**Quintile 2**	140	631	7.7	4.1	70.7	8.6%	69.3%
		(577 - 685)	(7.4 – 7.9)	(3.9 - 4.3)	(64.7 - 76.8)	(0.4 - 16.8)	(56.6 - 82.0)
**Quintile 3**	143	656	7.5	4.3	72.8	9.5%	65.8%
		(625 - 688)	(7.3 - 7.6)	(4.2 - 4.4)	(68.0 - 77.5)	(4.0 - 15.0)	(56.7 - 74.9)
**Quintile 4**	166	645	7.5	4.3	71.0	9.8%	66.2%
		(607 - 683)	(7.4 - 7.7)	(4.2 - 4.4)	(66.5 - 75.5)	(3.0 - 16.6)	(55.8 - 76.5)
**Least Deprived**	161	635	7.6	4.1	71.4	7.1%	65.8%
		(626 - 668)	(7.5 - 7.7)	(4.2 - 4.3)	(68.2 - 74.7)	(3.4 - 10.7)	(58.2 - 73.4)

Statistically significant difference between boys and girls, or across SIMD quintiles: *p<0.001
Figures are estimated marginal means or adjusted predicted probabilities and significance testing adjusted for season of measurement, mean wear time, and number of valid days.
‡Significance testing conducted as contrast between base category (most deprived) and all other quintiles

Prevalence estimates – Impact of changing analytical approach and adherence criteria

Both approaches produced significantly different estimates (p<0.05). Using the *daily* definition of adherence to assess guideline prevalence, approximately 11% of all children met the current recommendations. There was no significant difference between boys and girls. No statistically significant patterns were found by area deprivation, although a slightly higher proportion of those in the most deprived quintile met the guidelines (see Table 1). Although more boys met the guideline across all seasons except during autumn, there was no significant interaction (p=0.7) between gender and season (see Figure 1).

For peer review only - http://bmjopen.bmj.com/site/about/guidelines.xhtml

For peer review only

Using the *average* approach, 68% of children achieved the recommended level of activity, and significantly more boys met the guidelines than girls (see Table 1). No significant differences were evident between quintiles of deprivation. Contrary to the daily approach, there was a significant interaction between gender and season (p=0.047): a significantly higher proportion of boys met the 60-minute daily average in winter and autumn. However, a higher non-significant proportion of girls met the 60-minute daily average in summer compared to boys (see Figure 1).

Figure 2 illustrates the impact of varying the physical activity guideline reference point around 60 minutes. For both approaches, as expected, there was a linear increase in the proportion of children meeting the guidelines as the reference point was lowered from 70 minutes to 50 minutes. No significant differences existed between boys and girls at any reference point for the daily approach, although we observed a widening of the gap between them as the reference point was lowered. Conversely, there was a consistent significant difference between boys and girls across all levels when using the average approach, albeit with a tendency for the gap to reduce as the reference point was lowered.

INSERT FIGURE 1 HERE

INSERT FIGURE 2 HERE

For peer review only - http://bmjopen.bmj.com/site/about/guidelines.xhtml

For peer review only

Discussion

Statement of Principal Findings

This was the first representative study of objectively measured physical activity and sedentary levels among 10-11 year old children across Scotland. Using the daily measure (which required children to have at least 60 minutes of MVPA on every valid day of recording), only 11% of Scottish 10-11 year olds met the recommended guideline. However, this figure substantially increased to 68% when implementing the average measure (which required children to have at least 60 minutes daily MVPA on average across all valid days). Our results also indicated that reducing or increasing the 60-minute threshold by even as little as five minutes can substantially impact the prevalence rates. If the threshold was reduced to 55-minutes per day, we predict an increase in between 2-8 percentage points depending on sex and type of analysis (using an average or daily approach).

Strengths and limitations

This study was the first to investigate the objectively measured physical activity levels and guideline prevalence in a large, nationally representative, sample of 10-11 year old Scottish children. The data reduction protocol followed those of other large studies [6], allowing accurate comparisons to be made between this study and others, particularly with consideration to the cut points advocated in the literature to classify sedentary behaviour (<100 cpm) and MVPA (>2296cpm) [6]. A further strength of this study is the comparison of two approaches to the interpretation of guideline adherence, and assessment of the subsequent impact on interpretation of physical activity levels.

For peer review only - http://bmjopen.bmj.com/site/about/guidelines.xhtml

The study also had limitations. Waist mounted devices are typically poor at recording the acceleration associated with cycling or upper body dominant activities [9]. The devices were removed for water-based activities and contact sports so we will have underestimated these activities. Additionally, whilst our chosen cut points to classify MVPA were evidence based [19], other published cut points are available and their use would significantly alter our results.

Comparison with literature

The PA levels from this study are largely comparable to other large-scale, UK, objectively measured studies of PA in similar age groups (See Table 2).

Table 2 - Descriptive data from large, UK based, accelerometry studies using similar age groups

Study name	Setting	Year	Mean age (years)	Sample size	Mean CPM	Cut point for Sed/MVPA	Sedentary (hours)	MVPA (mins)	Prevalence of PA (%)
**SPACES**	Scotland wide	2015-2016	11.1	774	622	<100 >2296	7.5	72	11/68.3%
**MCS**	UK (Scotland Sample)	2008-2009	7.5	761	615	<100 >2241	6.4	62	52.5%
**GMS**	North East England	2008-2009	9.3	405	643	<1100 >3200	Not reported	24	5.7%
**SPEEDY**	South East England	2007	10.2	1,862	665	<100 >2000	7.6	74	70.5%
**ALSPAC**	South West England	2003-2005	11.8	5,595	580	<100 >3600	7.2	20	2.5%

For peer review only - http://bmjopen.bmj.com/site/about/guidelines.xhtml

SPACES: Studying Physical Activity in Children's Environments across Scotland
SPEEDY: Sport, Physical activity and Eating behaviour: Environmental Determinants in Young people [23]
ALSPAC: Avon Longitudinal Study of Parents and Children [5]
MCS: Millennium Cohort Study [10]
GMS: Gateshead Millennium Cohort Study [24]

The literature is relatively consistent in showing differences in physical activity levels between boys and girls and the recent publication by Cooper and colleagues [6], with over 6,000 9-10 year olds across 7 different countries, supports this. We did not find any statistically significant socioeconomic gradient in activity levels, although there was a tendency for those in the most deprived areas to engage in higher levels of total activity, MVPA, and less time sedentary than other quintiles of deprivation. Again, recent work from the Millennium Cohort study [10], and previous other large scale objective measured studies [5, 25] have shown little socioeconomic patterning at this age. It has been suggested, however, that active travel behaviours may be more prevalent among children from poorer neighbourhoods. They may, for example, be more likely to walk to school compared to children from more affluent neighbourhoods who are more likely to be driven to school [26]. This could explain the slightly higher levels of MVPA among children in more deprived neighbourhoods in our study. Future work with the SPACES dataset will explore this question. The seasonal variation evident in the present study was also consistent with previous work in the UK [27, 28]. Atkin and colleagues [29] for instance, found similar patterns across season but with boys consistently more active than girls when exploring time spent in MVPA. From an intervention, behaviour change, and policy perspective, it is vitally important to tailor our approaches to consider the differential effects of season on PA levels, particularly for girls. We plan to explore, using collected GPS data and Geographic Information

For peer review only - http://bmjopen.bmj.com/site/about/guidelines.xhtml

Systems (GIS) software, whether specific weather conditions and season influence the probability of missing the 60-minute threshold and therefore help to explain some of the discordance between the two approaches of guideline adherence.

Our most important finding is that either 11% or 68% of Scottish children meet the current UK guidelines, depending on the approach taken to measurement. This difference could be interpreted as either ‘quite poor’ or ‘relatively good’ and would have serious implications no matter which interpretation was taken. The only other study, known to the authors, who have published data using similar approaches, was conducted in Estonian children and young people (6-13 years old). Mooses and colleagues [12], using an identical MVPA ‘cut point’ (i.e. the Evenson cut point), identified a greater than two-fold increase in guideline prevalence dependent on approach (daily, 24% and average, 52%). However, the authors did not include weekend days, and daily adherence was classified as meeting 4 out 5 weekdays. The present study required a minimum of 4 weekdays and 1 weekend day to be included in analyses, and adherence to the daily approach was based on meeting each and every valid day of wear. Furthermore, the present study was specific to 10/11 year olds and nationally representative. Therefore, our results aren’t directly comparable. Previous studies have also identified the impact on prevalence estimates when using different ‘cut points’ to discern MVPA. The results from work by Griffiths and colleagues [10] for instance, indicated that the proportions of children meeting the PA guidelines changed from 84.0%/59.4% (boys and girls respectively) to 13.7%/0.4%, when altering their cut point from 2000cpm to 3000cpm. There have been calls for an international consensus process on accelerometry methodology

For peer review only - http://bmjopen.bmj.com/site/about/guidelines.xhtml

and reporting standards in youth, with a formal review of recommendations every 5 years to evaluate new science [18]. The recent creation of the International Children's Accelerometry Database [ICAD, 6] where standardised methods have re-analysed and re-integrated data from multiple international studies leading to cross-country comparisons is an important step in recognising the need to standardise measurement approaches.

The question remains however, which approach is more appropriate? Does activity 'every day' matter more, in health benefit terms, than the accumulation over the week? Is 'missing' one day out of one week problematic in terms of health [1, 30]? Furthermore, although the latest evidence demonstrates that meeting the 60 minutes of MVPA per day has positive health implications, particularly for adiposity and quality of life [13], there is little evidence on whether individuals whose activity levels approach, but do not reach, 60 minutes per day are less healthy, and whether narrowly falling short of 'every day' has implications for health outcomes [12]. We know that activity under the prescribed intensity of MVPA has substantial benefits on the maintenance of energy balance, i.e. Non-Exercise Activity Thermogenesis (NEAT) [31, 32]; evidence is growing of the benefits of light intensity activity on other aspects of health, such as the reduced risk of all-cause mortality [33], and the inverse association with type-2-diabetes risk factors (e.g. 2-h plasma glucose levels) in adults [34]. As far as the authors are aware, no literature exists on the optimal levels, and patterns, of physical activity intensities combined. Our results also highlight that the proportions of children meeting the UK guidelines vary considerably across 10 minutes either side of the 60-minute threshold. It would be

For peer review only - http://bmjopen.bmj.com/site/about/guidelines.xhtml

beneficial if studies elsewhere could investigate the health effects of differing patterns of daily MVPA (and sedentary and light PA) and the health related implications of changing the 60-minute threshold. Doing so may result in greater flexibility around guideline adherence, and will provide researchers with improved knowledge when communicating health messages to the public.

Implications for Policy

In 2014, Scotland launched the Active Scotland Outcomes Framework (ASOF), which set out ambitions for physical activity and sport in Scotland over the next 10 years. A range of indicators will track progress using SHeS data. Alongside the framework is a key national legacy 10 year programme designed to influence population levels of activity in adults and children [35]. The extent to which these initiatives are viewed as successful in increasing children and young people's physical activity will depend on the measures used to assess physical activity and we hope that our findings can inform the understanding of physical activity prevalence, as well as evaluation methods used to measure the impact of policy change. Griffiths and colleagues [10] were right to state that the clarity of cut points used for MVPA is vital for monitoring of policy objectives around physical activity but we also demonstrate here that the approach used to assess guideline adherence will determine the result and, therefore, the evaluation of interventions and policy objectives.

For peer review only - http://bmjopen.bmj.com/site/about/guidelines.xhtml

Acknowledgements

We would like to thank the children from the Growing up in Scotland longitudinal birth cohort study for taking part in the research and to members of the Scotcen Social Research team who assisted along the way.

Competing Interests

The authors declare no competing interests

Funding

This work was supported by the Medical Research Council [grant number MC_UU_12017/10] and Chief Scientist Office [grant number SPHSU10]; and the Scottish Government [grant number SR/SC 17/04/2012].

Author contribution statement

PM and AE ran the data collection process, and PM led on data processing and data analysis and drafted the article. RM and AE assisted with statistical analysis and interpretation of the data, and RM contributed to the graphical presentation of the results. AE and RM critically revised the article and all authors signed off the final draft.

Data Sharing Statement

We are committed to maximizing the use of SPACES study data to advance knowledge to improve young people's health, and welcome proposals for collaborative projects and data sharing. Our data sharing policy follows that of the Medical Research Council and aims to balance making data as widely and freely available as possible with safeguarding the privacy of participants, protecting confidential data, and maintaining the reputation of the study. Please contact Prof Anne Ellaway (anne.ellaway@glasgow.ac.uk) for further information.

For peer review only

For peer review only - http://bmjopen.bmj.com/site/about/guidelines.xhtml

For peer review only

References

1. Janssen, I. and A.G. Leblanc, *Systematic review of the health benefits of physical activity and fitness in school-aged children and youth.* Int J Behav Nutr Phys Act, 2010. **7**: p. 40.
2. Department of Health, *Start Active, Stay Active: a report on physical activity from the four home countries' Chief Medical Officers*, D.o. Health, Editor. 2011, Crown Copyright: London.
3. Hovald, P., *Chapter 7 Physical activity*, in *The Scottish Health Survey 2015 edition: volume1, main report. A National Statistics Publication for Scotland*, D. Campbell-Jack, S. Hinchliffe, and L. Rutherford, Editors. 2016, Scottish Government: Edinburgh.
4. Basterfield, L., et al., *Surveillance of physical activity in the UK is flawed: validation of the Health Survey for England Physical Activity Questionnaire.* Arch Dis Child, 2008. **93**(12): p. 1054-8.
5. Riddoch, C.J., et al., *Objective measurement of levels and patterns of physical activity.* Arch Dis Child, 2007. **92**(11): p. 963-9.
6. Cooper, A.R., et al., *Objectively measured physical activity and sedentary time in youth: the International children's accelerometry database (ICAD).* Int J Behav Nutr Phys Act, 2015. **12**: p. 113.
7. Konstabel, K., et al., *Objectively measured physical activity in European children: the IDEFICS study.* Int J Obes (Lond), 2014. **38 Suppl 2**: p. S135-43.
8. Welk, G.J., C.B. Corbin, and D. Dale, *Measurement issues in the assessment of physical activity in children.* Res Q Exerc Sport, 2000. **71**(2 Suppl): p. S59-73.
9. Corder, K., et al., *Assessment of physical activity in youth.* J Appl Physiol (1985), 2008. **105**(3): p. 977-87.
10. Griffiths, L.J., et al., *How active are our children? Findings from the Millennium Cohort Study.* BMJ Open, 2013. **3**(8): p. e002893.
11. World Health Organization, W., *Health Behaviour in School-Aged Children (HBSC) Study: International Report from the 2013/2014 Survey*, W.-. Europe, Editor. 2016, World Health Organisation: Copenhagen, Denmark.
12. Mooses, K., et al., *Different Methods Yielded Two-Fold Difference in Compliance with Physical Activity Guidelines on School Days.* PLoS One, 2016. **11**(3): p. e0152323.
13. Poitras, V.J., et al., *Systematic review of the relationships between objectively measured physical activity and health indicators in school-aged children and youth.* Appl Physiol Nutr Metab, 2016. **41**(6 Suppl 3): p. S197-239.
14. Tremblay, M.S., et al., *New Canadian physical activity guidelines.* Appl Physiol Nutr Metab, 2011. **36**(1): p. 36-46; 47-58.
15. Robusto, K.M. and S.G. Trost, *Comparison of three generations of ActiGraph activity monitors in children and adolescents.* J Sports Sci, 2012. **30**(13): p. 1429-35.
16. Romanzini, M., et al., *Calibration of ActiGraph GT3X, Actical and RT3 accelerometers in adolescents.* Eur J Sport Sci, 2014. **14**(1): p. 91-9.

For peer review only - http://bmjopen.bmj.com/site/about/guidelines.xhtml

17. Rowlands, A.V., E.L. Pilgrim, and R.G. Eston, *Patterns of habitual activity across weekdays and weekend days in 9-11-year-old children.* Prev Med, 2008. **46**(4): p. 317-24.
18. Cain, K.L., et al., *Using accelerometers in youth physical activity studies: a review of methods.* J Phys Act Health, 2013. **10**(3): p. 437-50.
19. Evenson, K.R., et al., *Calibration of two objective measures of physical activity for children.* J Sports Sci, 2008. **26**(14): p. 1557-65.
20. Trost, S.G., et al., *Comparison of accelerometer cut points for predicting activity intensity in youth.* Med Sci Sports Exerc, 2011. **43**(7): p. 1360-8.
21. Government, S., *Scottish Index of Multiple Deprivation: SIMD16 Technical Notes.* 2016, National Statistics.
22. Bradshaw, P., et al., *Growing Up In Scotland Sweep 1 - 2005 User Guide*. 2005, Scottish Centre for Social Research: Edinburgh.
23. Steele, R.M., et al., *Targeting sedentary time or moderate- and vigorous-intensity activity: independent relations with adiposity in a population-based sample of 10-y-old British children.* Am J Clin Nutr, 2009. **90**(5): p. 1185-92.
24. Basterfield, L., et al., *Longitudinal study of physical activity and sedentary behavior in children.* Pediatrics, 2011. **127**(1): p. e24-30.
25. van Sluijs, E.M., et al., *Physical activity and dietary behaviour in a population-based sample of British 10-year old children: the SPEEDY study (Sport, Physical activity and Eating behaviour: environmental Determinants in Young people).* BMC Public Health, 2008. **8**: p. 388.
26. GCPH, *Children's travel to school – are we moving in the right direction*, G.C.f.P. Health, Editor. 2011, GCPH: Glasgow.
27. Rich, C., L.J. Griffiths, and C. Dezateux, *Seasonal variation in accelerometer-determined sedentary behaviour and physical activity in children: a review.* Int J Behav Nutr Phys Act, 2012. **9**: p. 49.
28. Atkin, A.J., et al., *Seasonal Variation in Children's Physical Activity and Sedentary Time.* Med Sci Sports Exerc, 2016. **48**(3): p. 449-56.
29. Atkin, A.J., et al., *Seasonal Variation in Children's Physical Activity and Sedentary Time.* Medicine and Science in Sports and Exercise, 2016. **48**(3): p. 449-456.
30. Bull, F. and E.W. Group, *Physical Activity Guidelines in the U.K.: Review and Recommendations.* 2010, Loughborough University: School of Sport, Exercise and Health Sciences, Loughborough University.
31. Levine, J.A., *Nonexercise activity thermogenesis--liberating the life-force.* J Intern Med, 2007. **262**(3): p. 273-87.
32. Katzmarzyk, P.T., *Physical activity, sedentary behavior, and health: paradigm paralysis or paradigm shift?* Diabetes, 2010. **59**(11): p. 2717-25.
33. Loprinzi, P.D., *Light-Intensity Physical Activity and All-Cause Mortality.* Am J Health Promot, 2016.
34. Healy, G.N., et al., *Objectively measured light-intensity physical activity is independently associated with 2-h plasma glucose.* Diabetes Care, 2007. **30**(6): p. 1384-9.
35. Scottish Government, *Physical Activity Implementation Plan: A More Active Scotland*. 2014, Scottish Government.

For peer review only - http://bmjopen.bmj.com/site/about/guidelines.xhtml

Figure Legends

Figure 1- Physical activity prevalence across season of measurement by sex and measurement approach

*p<0.01; statistically significant difference between boys and girls within season

‡ Statistically significant interaction between gender and season, p<0.05

Figures are predicted probabilities and significance testing is adjusted for mean wear time, and number of valid days.

Figure 2 - Physical activity prevalence by sex and threshold classification reference – daily and average approaches

*p<0.01, **p<0.001; statistically significant difference between boys and girls within threshold reference point and measurement approach

Figures are predicted probabilities and significance testing is adjusted for season of measurement, mean wear time, and number of valid days.

For peer review only

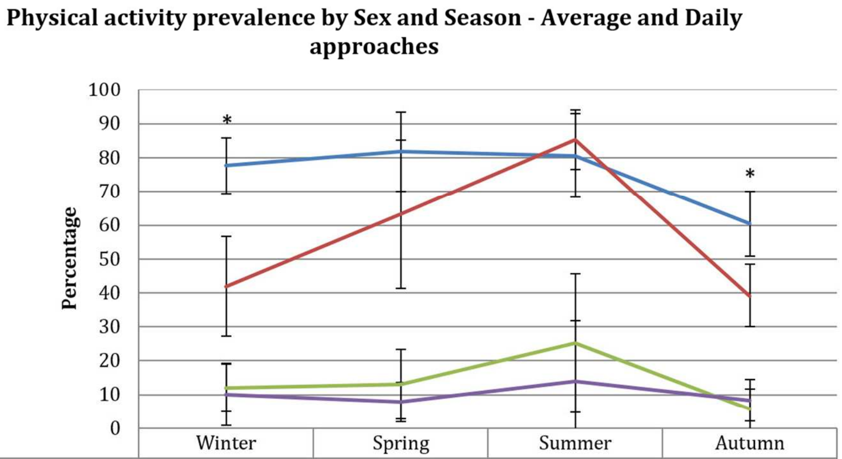

	Winter	Spring	Summer	Autumn
Male - average ‡	78	82	81	60
Female - average ‡	42	63	85	39
Male - daily	12	13	25	6
Female - daily	10	8	14	8

Figure 1- Physical activity prevalence across season of measurement by sex and measurement approach
*p<0.01; statistically significant difference between boys and girls within season
‡ Statistically significant interaction between gender and season, p<0.05
Figures are predicted probabilities and significance testing is adjusted for mean wear time, and number of valid days.

115x73mm (300 x 300 DPI)

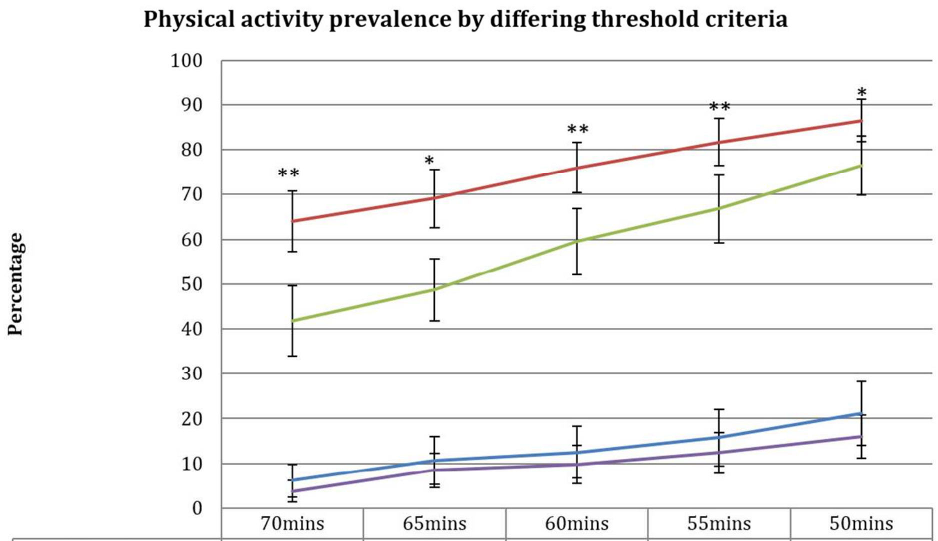

	70mins	65mins	60mins	55mins	50mins
Male - Average	64	69	76	82	86
Female - Average	42	49	59	67	77
Male - Daily	6	11	12	16	21
Female - Daily	4	9	10	12	16

Figure 2 - Physical activity prevalence by sex and threshold classification reference – daily and average approaches
*p<0.01, **p<0.001; statistically significant difference between boys and girls within threshold reference point and measurement approach
Figures are predicted probabilities and significance testing is adjusted for season of measurement, mean wear time, and number of valid days.

106x73mm (300 x 300 DPI)

Supplementary Table 1: Comparison of weighted sample to known national level SES/demographic distributions

Demographic variable	SPACES weighted sample distribution	Sweep 8 Growing up in Scotland (GUS) weighted sample distribution
Income (per annum)		
<3,999 - £9,999	3%	5%
£10,000 - £19,999	20%	21%
£20,000 - £28,999	18%	16%
£29,000 - £37,999	14%	14%
£38,000 - £49,999	14%	15%
>50,000	27%	29%
Mothers age at birth (years)		
Under 20	4%	7%
20 -29	43%	41%
30 – 39	50%	49%
40 or older	3%	3%
Marital status		
Married	61%	68%
Cohabiting	19%	15%
Single	11%	9%
Widowed	1%	1%
Divorced	5%	3%
Separated	3%	4%
SIMD quintile (2012)		
Most	21%	20%
2nd	18%	21%
Middling	19%	20%
4th	21%	18%
Least Deprived	21%	21%
Highest educational qualification in household		
No qualification	3%	6%
Lower level Standard Grades or equivalent	3%	4%
Upper level Standard Grades or equivalent	19%	19%
Higher Grades or equivalent	39%	33%
Degree level academic or equivalent	35%	38%
Other	1%	0.4%
Urban/Rural dwelling		
Large urban	36%	38%
Other Urban	33%	32%
Small accessible towns	9%	10%
Small remote towns	3%	3%
Accessible rural	12%	13%
Remote rural	7%	4%
BMI UK categories		
Underweight	2%	2%
Healthy weight	64%	64%
Overweight	18%	15%
Obese	16%	19%

For peer review only

STROBE Statement—checklist of items that should be included in reports of observational studies

	Item No	Recommendation
**Title and abstract**	1	(*a*) Indicate the study's design with a commonly used term in the title or the abstract ☑ page 2
		(*b*) Provide in the abstract an informative and balanced summary of what was done and what was found ☑ page 2
**Introduction**		
Background/rationale	2	Explain the scientific background and rationale for the investigation being reported ☑ Page 4/5/6
Objectives	3	State specific objectives, including any prespecified hypotheses ☑ Page 6
**Methods**		
Study design	4	Present key elements of study design early in the paper ☑ Page 7
Setting	5	Describe the setting, locations, and relevant dates, including periods of recruitment, exposure, follow-up, and data collection ☑ Page 7/8/9
Participants	6	(*a*) *Cohort study*—Give the eligibility criteria, and the sources and methods of selection of participants. Describe methods of follow-up<br>*Case-control study*—Give the eligibility criteria, and the sources and methods of case ascertainment and control selection. Give the rationale for the choice of cases and controls<br>*Cross-sectional study*—Give the eligibility criteria, and the sources and methods of selection of participants ☑ Page7/8
		(*b*) *Cohort study*—For matched studies, give matching criteria and number of exposed and unexposed<br>*Case-control study*—For matched studies, give matching criteria and the number of controls per case
Variables	7	Clearly define all outcomes, exposures, predictors, potential confounders, and effect modifiers. Give diagnostic criteria, if applicable ☑ Page 9
Data sources/ measurement	8*	For each variable of interest, give sources of data and details of methods of assessment (measurement). Describe comparability of assessment methods if there is more than one group ☑ Page 9
Bias	9	Describe any efforts to address potential sources of bias ☑ Page 7/9
Study size	10	Explain how the study size was arrived at ☑ Page 7
Quantitative variables	11	Explain how quantitative variables were handled in the analyses. If applicable, describe which groupings were chosen and why ☑ Page 9/10
Statistical methods	12	(*a*) Describe all statistical methods, including those used to control for confounding ☑Page 10
		(*b*) Describe any methods used to examine subgroups and interactions ☑ Page 10
		(*c*) Explain how missing data were addressed
		(*d*) *Cohort study*—If applicable, explain how loss to follow-up was addressed<br>*Case-control study*—If applicable, explain how matching of cases and controls was addressed<br>*Cross-sectional study*—If applicable, describe analytical methods taking account of sampling strategy ☑ Page 10
		(*e*) Describe any sensitivity analyses

Continued on next page

For peer review only

For peer review only - http://bmjopen.bmj.com/site/about/guidelines.xhtml

**Results**		
Participants	13*	(a) Report numbers of individuals at each stage of study—eg numbers potentially eligible, examined for eligibility, confirmed eligible, included in the study, completing follow-up, and analysed ☑ Page 7
		(b) Give reasons for non-participation at each stage ☑ Page 7
		(c) Consider use of a flow diagram
Descriptive data	14*	(a) Give characteristics of study participants (eg demographic, clinical, social) and information on exposures and potential confounders ☑ Page 7
		(b) Indicate number of participants with missing data for each variable of interest
		(c) *Cohort study*—Summarise follow-up time (eg, average and total amount)
Outcome data	15*	*Cohort study*—Report numbers of outcome events or summary measures over time
		Case-control study—Report numbers in each exposure category, or summary measures of exposure
		Cross-sectional study—Report numbers of outcome events or summary measures ☑ Page 11
Main results	16	(*a*) Give unadjusted estimates and, if applicable, confounder-adjusted estimates and their precision (eg, 95% confidence interval). Make clear which confounders were adjusted for and why they were included ☑ Page 11/12/13
		(*b*) Report category boundaries when continuous variables were categorized
		(*c*) If relevant, consider translating estimates of relative risk into absolute risk for a meaningful time period
Other analyses	17	Report other analyses done—eg analyses of subgroups and interactions, and sensitivity analyses
**Discussion**		
Key results	18	Summarise key results with reference to study objectives ☑ Page 13
Limitations	19	Discuss limitations of the study, taking into account sources of potential bias or imprecision. Discuss both direction and magnitude of any potential bias ☑ Page 14
Interpretation	20	Give a cautious overall interpretation of results considering objectives, limitations, multiplicity of analyses, results from similar studies, and other relevant evidence ☑ Page 17/18/19
Generalisability	21	Discuss the generalisability (external validity) of the study results ☑ Page 14/15/16
**Other information**		
Funding	22	Give the source of funding and the role of the funders for the present study and, if applicable, for the original study on which the present article is based ☑ Page 19

*Give information separately for cases and controls in case-control studies and, if applicable, for exposed and unexposed groups in cohort and cross-sectional studies.

Note: An Explanation and Elaboration article discusses each checklist item and gives methodological background and published examples of transparent reporting. The STROBE checklist is best used in conjunction with this article (freely available on the Web sites of PLoS Medicine at http://www.plosmedicine.org/, Annals of Internal Medicine at http://www.annals.org/, and Epidemiology at http://www.epidem.com/). Information on the STROBE Initiative is available at www.strobe-statement.org.

For peer review only
